# Editorial: *Antibiotics* Special Issue on Pharmacokinetic/Pharmacodynamic Models of Antibiotics

**DOI:** 10.3390/antibiotics11111540

**Published:** 2022-11-03

**Authors:** Razieh Kebriaei, Andrew D. Berti

**Affiliations:** 1 Department of Outcomes and Translational Sciences, College of Pharmacy, The Ohio State University, Columbus, OH 43210, USA; 2 Department of Pharmacy Practice, Eugene Applebaum College of Pharmacy and Health Sciences, Wayne State University, Detroit, MI 48201, USA

Pharmacokinetic/pharmacodynamic (PK/PD) modeling is an essential tool for rational drug development and treatment design [1]. These in vitro or in silico surrogates simulate the clearance of medications over time in virtual patients, while quantifying the relationships between dose, concentration, and drug effects. These models allow for clinical outcome predictions of untested regimens and optimized treatment selection based on complex patient variables and rational quantified results [2]. In vitro studies offer multiple flexibilities, such as the duration of the PK/PD model, the amount of bacterial load, and the variability of dose/treatment regimens at both lower costs and reduced risk to patients. We promote PK/PD models of antibiotics to accelerate the selection of optimized treatment regimens and the development of new therapies. This Special Issue includes seven submissions with a focus on PK/PD models, as described below.

The contribution from Golikova et al. [3] applied traditional one-compartment modeling to determine mutant prevention concentrations of anti-staphylococcal antibiotics when given in combination. Antimicrobial resistance development is a significant global issue and can limit the effectiveness of infectious disease treatments. The authors apply the one-compartment in vitro dynamic bioreactor model pioneered by Blaser et al. [4] to determine if the presence of a secondary antibiotic (gentamicin) can alter the mutant prevention concentration of the primary antibiotic (daptomycin) against the human pathogen *Staphylococcus aureus*. The authors determined that mutant prevention concentrations (MPCs) determined in antibiotic combination were significantly lower than monotherapy-only MPCs, i.e., when assessed at a ratio of each antibiotic’s respective 24 h area under the concentration–time curve (AUC). They defined time > MPC as a predictor of an antibiotic in terms of antimutant efficacy. These findings support a role for antimicrobial combinations against this important pathogen, not just for enhanced pharmacodynamic effects but also for the suppression of resistance development.

Innovative applications of mathematical and experimental modeling were also applied to important enteric pathogens. Smith et al. [5] reported their findings based on examining how polymyxin B exposure modifies the population dynamics of *Escherichia coli* with different *mcr-1* carriage prevalence. Specifically, the authors found that even in populations with a low number of *mcr-1*-positive cells, the simulated exposure of polymyxin B rapidly results in the expansion of the *mcr-1-*positive subpopulation and resistance to the intervention. This finding persisted in a 5000-patient simulation which incorporated the pharmacokinetic variability of polymyxin B in critically ill patients. Even front-loading polymyxin B doses was insufficient to prevent regrowth. This combination of mathematical modeling and in vitro experiments makes a strong argument for polymyxin combination therapy rather than monotherapy when even a minor subpopulation of *mcr-1* carriage is suspected.

Although the one-compartment bioreactor model is a powerful tool used to incorporate the dynamics of antibiotic exposure, there are additional valuable strategies available to assess antimicrobial pharmacodynamics. Gubenšek et al. [6] performed time–kill assays against three strains of *Neisseria gonorrhoeae.* Specifically, they applied the Regoes mathematical model [7] to determine growth rates, pharmacodynamic MICs (zMICs), and the Hill coefficient trained by estimates from non-linear regression models. The authors assessed the antimicrobial activity of ceftriaxone, ertapenem, fosfomycin, and gentamicin paralleling agents in a recent clinical trial. While the clinical trial demonstrated that ertapenem was non-inferior to ceftriaxone, the same did not apply to fosfomycin or gentamicin. Interestingly, Gubenšek et al. report superior bacterial killing with high-dose ceftriaxone even against ceftriaxone-resistant isolates compared to killing by ertapenem. They also report a better correspondence between zMIC than traditional minimum inhibitory concentration (MIC) with regard to bacterial killing, particularly with fosfomycin. Indeed, the authors suggest that the determination of these additional PD parameters may better predict true PK/PD indices.

Further evidence supporting the utility of different PD parameters in predicting clinical outcomes is provided by Zhou et al. [8]. These investigators examined the probability of target attainment based on time above the MIC for cefoperazone–sulbactam in the treatment of *Acinetobacter baumannii*. They performed a prospective open-label study enrolling 54 patients and determined individual PK and PD indices for each of the two antibiotic components. The time above the MIC was identified as the most predictive index for each medication, as expected. However, the authors further identified that a composite product of time above the MIC for both components outperformed the time above the MIC for either individual component. Using population PK values and Monte Carlo simulations, the authors suggest the probability of clinical and microbiological cure rates under various combinations of dosing regimens and organism MICs.

Structural PK models are useful tools to forecast the probability of target attainment, but post-deployment validation and sensitivity testing are inconsistently performed. In this collection, Kim et al. [9] use Monte Carlo simulations to assess the relative bias and relative root mean square error of popular one-compartment and two-compartment models of vancomycin pharmacokinetics. The authors demonstrate that models with fewer compartments and more sparse sampling result in inaccurate and imprecise vancomycin PK profile estimates. This is of particular concern as clinicians performing vancomycin pharmacokinetic estimates often utilize single-compartment PK models and infrequent sampling to determine appropriate dosing.

While an analysis of clinical isolates provides valuable information, an examination of environmental isolates may provide a more comprehensive picture of antimicrobial resistance traits. The next contribution to this collection simulated the impact of combination antibiotic exposure on enterobacteriaceae collected from hospital or water treatment plant wastewater. The authors restricted analysis to strains possessing horizontal gene transfer elements to enrich organisms with mobile antibiotic resistance traits. Using checkerboard methodology [10], Fadare et al. [11] identify combinations that demonstrated synergy and validated those preliminary findings in time–kill curves. Consistently, the simulated combinations outperformed single-agent therapy, resulting in sustained killing to the limit of detection. Since resistance traits are often linked and can travel horizontally on the same integron, identifying which antibiotic combinations retain activity in the presence of common horizontal gene transfer elements provides a significant contribution to the antimicrobial synergy literature.

The final contribution to this collection is provided by Parra et al. [12]. These authors examine the probability of target attainment (PTA) for vancomycin in patients with febrile neutropenia. A cohort of 14 patients was used to train a population PK model, including multiple patient-specific covariates. This PK model was used to evaluate the PTA for multiple PD outcomes, including the historical minimum steady state serum concentration (C_ss min_) of 15–20 mg/L and the updated target of 24 h area under the concentration-time curve (AUC_24_)/MIC > 400. Following training and model reduction through the use of an artificial neural network, a test cohort of n = 600 simulated patients evaluated which input variables had the most impact on target attainment. For the AUC_24_/MIC > 400 target, the driving factor was the organism MIC. In contrast, targets including an AUC of 400–600 and a C_ss min_ of 15–20 mg/L were more dependent on the daily dose and patient estimated glomerular filtration rate, although additional factors including infusion time and dosing interval remained significant PTA drivers for the C_ss min_ target. This questions the appropriateness of a C_ss min_ target as a surrogate for the more accepted AUC target. Furthermore, this work suggests that vancomycin PK is comparable in patients with febrile neutropenia requiring a 25% increase in daily dose at most, as compared to vancomycin PK in other populations.

The reports in this Special Issue provide a comprehensive analysis of the complex interactions between PK and PD parameters. These models are applied to evaluate different antibiotic treatments, optimize dosage regimens, and prevent resistance development. Standard parameters, such as the area under a concentration–time curve (AUC), the MIC, the time above the MIC and AUC/MIC, help with analyzing the efficacy of antibiotics using quantitative methods. In this context, however, the additional optimization of PK/PD indices is required due to multiple mechanisms of action in antibiotics, differences in combination therapy versus monotherapy, and unpredictable mutagenesis/resistance profiles in pathogens [13,14]. We strongly believe that the one-parameter-fits-all approach is not sufficient to capture the inherently variable nature of antibiotic–pathogen interactions. Optimizing PK/PD indices helps to better dissect pathogenic behavior during exposure to antibiotics. Mutant prevention concentration (MPC), zMIC, multiple parameter-based PD models, and kill rate-based PK/PD models provide additional insight compared to standard PK/PD parameters. The optimization of PK/PD parameters will help to not only ensure antimicrobial effectiveness, but also to prevent resistance development and avoid high-dose side effects.
